# Affinity purification of human m-calpain through an intrinsically disordered inhibitor, calpastatin

**DOI:** 10.1371/journal.pone.0174125

**Published:** 2017-03-20

**Authors:** Hung Huy Nguyen, Mihaly Varadi, Peter Tompa, Kris Pauwels

**Affiliations:** 1 VIB Center for Structural Biology (CSB), Vlaams Instituut voor Biotechnologie (VIB), Brussels, Belgium; 2 Structural Biology Brussels (SBB), Vrije Universiteit Brussel, Brussels, Belgium; 3 Institute of Enzymology, Research Centre for Natural Sciences of the Hungarian Academy of Sciences, Budapest, Hungary; Russian Academy of Medical Sciences, RUSSIAN FEDERATION

## Abstract

Calpains are calcium-activated proteases that have biomedical and biotechnological potential. Their activity is tightly regulated by their endogenous inhibitor, calpastatin that binds to the enzyme only in the presence of calcium. Conventional approaches to purify calpain comprise multiple chromatographic steps, and are labor-intensive, leading to low yields. Here we report a new purification procedure for the human m-calpain based on its reversible calcium-mediated interaction with the intrinsically disordered calpastatin. We exploit the specific binding properties of human calpastatin domain 1 (hCSD1) to physically capture human m-calpain from a complex biological mixture. The dissociation of the complex is mediated by chelating calcium, upon which heterodimeric calpain elutes while hCSD1 remains immobilized onto the stationary phase. This novel affinity-based purification was compared to the conventional multistep purification strategy and we find that it is robust, it yields a homogeneous preparation, it can be scaled up easily and it rests on a non-disruptive step that maintains close to physiological conditions that allow further biophysical and functional studies.

## Introduction

Calpains (EC 3.4.22.53) are intracellular calcium-activated cysteine proteases with broad endopeptidase specificity. Members of the calpain family have been identified in a wide range of eukaryotic organisms and even in some bacteria [1,2]. In Mammalia, these proteins are reported to be involved in various key physiological processes such as cell mobility, embryogenesis, apoptosis and various signal transduction pathways [1]. So far, 15 human genes are known to encode calpain-like protease domains [2]. The best studied calpain isoforms are the ubiquitously expressed μ-calpain and m-calpain, which require micromolar and millimolar concentrations of calcium for their enzymatic activation, respectively. X-ray crystallographic analyses have identified their unique activation mechanism: they are heterodimers consisting of an 80kDa large subunit (consisting of 4 domains) and a 30kDa small subunit (consisting of 2 domains) that undergo major conformational rearrangements upon binding multiple calcium ions [3,4]. Since calpain activity is involved in various pathophysiological phenomena, the regulation of calpain is crucial for proper cell functioning. Besides calcium, calpain activity is regulated by an endogenous inhibitor, calpastatin that can only bind to the calcium-activated conformation. Calpastatin is an intrinsically disordered protein (IDP) composed of four inhibitory domains, each capable of inhibiting the enzyme [5]. Each inhibitory domain consists of 3 subdomains (region A, B and C) that only acquire a stable conformation upon binding to the calcium-activated calpain surface.

Increased intracellular calcium levels lead to hyperactivation of calpain and are associated with a large variety of pathologies: m-calpain dysregulation is tightly associated with human diseases such as cancer, cataract and neurodegenerative syndromes like Alzheimer’s disease [6]. Calpains are thus of great medical and therapeutic interest, and there is a need for calpain-specific inhibitors that will not affect other cysteine proteases like cathepsin B and cathepsin L.

To better understand the structure-function relationship and to implement that knowledge to design efficient drugs to cure the diseases, it is necessary to produce calpain at sufficient quantity and quality. Active human and rat μ-calpain have been recombinantly expressed and purified using *Escherichia coli* and the baculovirus expression systems [7,8]. Recombinant rat m-calpain has been produced on large scale in *E*. *coli* [9,10], while a recombinant version of human m-calpain has been successfully expressed in *E*. *coli* by co-expressing the hexahistidine-tagged large subunit with a truncated small subunit [11]. Despite the application of affinity-tags to facilitate the specific isolation of these proteins, the methodology to purify them is time consuming as it consists of multiple chromatographic steps, which compromises the protein yield. To address the problems, several affinity-based chromatographic methods utilizing immobilized substrates (e.g. casein), inhibitors and antibodies have been reported [7,12–15]. Still, these methods either have the risk of co-purifying of proteins that show calcium-dependent binding to the immobilized substrates or require pre-chromatographic steps in the sample preparation[9,14,16]. The most recently reported purification strategy for recombinant human m-calpain requires four chromatographic steps to obtain pure and active enzyme [11].

Here, we describe the development of a single-step affinity chromatography strategy by exploiting the binding properties of the intrinsically disordered human calpastatin domain 1 (hCSD1) to isolate recombinantly produced human m-calpain and selected variants thereof from *E*. *coli* lysate.

## Materials and methods

### Expression vectors and mutagenesis

The expression plasmids of the wild type (WT) 80kDa human m-calpain large subunit (CAPN2 in pET24b(+)) and the truncated 23kDa small subunit (CAPNS1ΔG2 in pACpET) were generously provided by Dr. Hiroyuki Sorimachi (Tokyo Metropolitan Institute of Medical Science, Japan)[11]. An inactive m-calpain mutant large subunit, in which the cysteine at position 105 was mutated to alanine (C105A), was generated from the wild type with the QuickChange Site-Directed Mutagenesis kit (Agilent Technologies) using oligonucleotides 5’-ggagccctgggtgacgcctggctgctggcagcc-3’ and 5’-ggctgccagcagccaggcgtcacccagggctcc-3’ (the C105A nucleotide substitution is underlined). The successful mutagenesis was confirmed through DNA sequencing (VIB genetic sequencing facility). The expression plasmid of the human calpastatin domain 1 (hCSD1; corresponding to region A137-K277 of the human calpastatin amino acid sequence) was obtained from Dr. Masatoshi Maki (Institute for Virus Research, Japan)[17]. An N-terminal glutathione S-transferase-tagged (GST-tagged) version of hCSD1 was expressed from a synthetic gene that was constructed in the pGEX-4T-1 (GE Healthcare Life Science) vector by GeneArt AG (Life Technologies).

### Expression of the Wild Type (WT) and inactive mutant human m-calpain

The large and small subunit of WT human m-calpain were co-expressed in *Escherichia coli* BL21(DE3) (Invitrogen)[11]. The bacterial cells were grown in 2L baffled flasks at 27°C and 180 rpm in a minitron incubator shaker (Infors-HT) until the optical density at 600nm (OD_600_) reached 0.6 to 0.8. The protein expression was induced by the addition of 0.2mM isopropyl β-D-1-thiogalactopyranoside (IPTG) and continued growth for 6h at 22°C while shaking at 180rpm.

The C105A variant of the large subunit was co-expressed with the truncated small subunit in *E*. *coli* BL21(DE3) and the cells were initially grown at 30°C and 180 rpm shaking until the OD_600_ reached 0.3. The temperature was then changed to 16°C while shaking at 180 rpm until the OD_600_ reached 0.6 to 0.8. The protein expression was induced by adding 0.25mM IPTG and the induced cells were incubated for 16h at 16°C while shaking at 180 rpm.

The C105A large subunit of m-calpain was expressed in *E*. *coli* BL21(DE3). The bacterial culture was initially grown at 30°C, 180rpm until OD_600_ reached 0.6. The protein expression was induced by adding 0.2mM IPTG and continued growth at 16°C for only 6h while shaking at 180 rpm.

### Production of hCSD1 and its variations

The hCSD1 expression plasmid was transformed into the *E*. *coli* BL21-AI strain (Invitrogen). The transformants were grown in ampicillin-containing (100 μg/mL) Luria-Bertani broth (LB) at 37°C with vigorous shaking at 180 rpm. The protein expression was induced with 1mM IPTG and 0.2% L-arabinose at OD_600_ of ~0.5 to 0.8, and continued shaking for 4 hours a 180 rpm, 37°C.

The induced cells were harvested and centrifuged at 5000g for 15 minutes. The pellets were re-suspended in lysis buffer containing 20mM Tris-Cl pH 7.5, 0.1mM ethylenediaminetetraacetic acid (EDTA), 25mM NaCl, 2mM 1,4-dithiothreitol (DTT), 2mM 4-(2-aminoethyl)benzenesulfonyl fluoride hydrochloride (AEBSF) and 5mM benzamidine. The suspended cells were heated for 10 minutes at 99°C, followed by centrifugation at 21191g for 45 minutes. The supernatant was filtered via a 0.22μm polyethersulfone membrane and applied to a Hitrap DEAE FF anion exchange column (GE Healthcare) equilibrated with buffer containing 20mM Tris-Cl pH 7.5, 0.1mM EDTA, 2mM DTT at flow rate of 1ml/min. hCSD1 was eluted from the column using a step elution of 150mM NaCl for 10 column volumes in 20mM Tris-Cl pH 7.5, 0.1mM EDTA, 2mM DTT. The purified protein was applied to a Superdex 75 gel filtration column equilibrated with 30mM 3-(N-morpholino)propanesulfonic acid (MOPS) pH 7.5, 1mM tris(2-carboxyethyl)phosphine (TCEP).

The GST-tagged hCSD1 was expressed in *E*.*coli* BL21(DE3) at 37°C. The protein expression was induced with 1mM IPTG at an OD_600_ of 0.6–0.8, followed by 4 hours at 37°C and 180rpm. The cells were harvested by centrifugation (at 5000g for 15 minutes) and re-suspended in phosphate-buffered saline (PBS) supplemented with Complete^®^ protease inhibitor tablets (Roche Diagnostics), followed by a pulse sonication (2 seconds on, 5 seconds off) for 2 minutes (60% amplitude) using 130Watt Ultrasonic Processor (Sonics & Materials, INC.). The suspended cells were centrifuged at 21191g for 30 minutes. The lysate was filtered (0.22μm polyethersulfone membrane) and applied to a GSTrap FF column (GE Healthcare) equilibrated with PBS. The protein was eluted by a step elution of 10 mM reduced glutathione in 50 mM Tris-Cl pH 8.0.

The biotinylation of hCSD1 was accomplished using EZ-Link^™^ NHS-PEG_4_-Biotinylation kit (Pierce Biotechnology) according to the manufacturer’s instructions. The integrity of the biotinylated sample was verified by SDSPAGE analysis. To quantify the extent of the biotinylation we used the absorbance-based 2-(4′-hydroxyazobenzene) benzoic acid (HABA) dye assay that was provided by the manufacturer of the biotinylation kit (Pierce Biotechnology).

### Conventional purification of calpain

The established purification procedure for recombinantly produced human m-calpain comprises several chromatographic steps and was performed as described by Hata et al. (2012) with some minor modifications as follows: (1) 0.3 mM AEBSF was used as a protease inhibitor in the lysis buffer instead of 0.3 mM PMSF; (2) the DEAE-toyopearl and the MonoQ HR10/10 anion exchange columns were substituted by a Hitrap DEAE FF and a MonoQ 4.6/100 PE anion exchange columns (GE Healthcare), respectively.

### Affinity purification (AF) of calpain

All purification procedures were performed at 4°C. The induced cells were harvested and centrifuged at 5000g, 15 minutes. The pellet was re-suspended in lysis buffer containing 30mM MOPS pH7.5, 1mM EDTA, 1mM TCEP and supplemented with Roche protease inhibitors cocktail (which does not contain irreversible Cys-protease inhibitors). The cells were lysed by sonication using a series of short pulse (2 seconds on-5 seconds off, 80% amplitude) with 130Watt Ultrasonic Processor (Sonics & Materials, INC.). Then we loaded 2 mg of biotinylated hCSD1 (b-hCSD1) onto a 1ml Streptavidin HP column (GE Healthcare) equilibrated with 30mM MOPS pH7.5, 1mM TCEP at the flow rate of 0.1 ml/min using an Akta Avant (GE Healthcare). The b-hCSD1-immobilized column (affinity column) was washed with buffer containing 30mM MOPS pH7.5, 200mM NaCl, 5mM CaCl_2_, 1mM TCEP for 5 column volumes. The calpain lysate was supplemented with 200mM NaCl and 5mM CaCl_2_ immediately before applying it to the affinity column at the flow rate of 0.5ml/min. The column was washed with buffer containing 30mM MOPS pH7.5, 200mM NaCl, 5mM CaCl_2_, 1mM TCEP and the protein was eluted by a step elution with 20mM MOPS pH 8.0, 1mM TCEP and 10mM EDTA.

### Enzymatic calpain activity assay

The proteolytic activity of WT m-calpain and inactive C105A-calpain was monitored by using the fluorogenic substrate N-succinyl-Leu-Leu-Val-Tyr-AMC (ENZO Life Sciences). The lyophilized substrate was dissolved in 100% dimethyl sulfoxide (DMSO) and diluted in reaction mixture (200μl in total volume) containing 20mM MOPS, 1mM TCEP, and 3mM CaCl_2_ to a series of concentrations from 4 to 80 μM. At last, 150nM enzyme was added to the reaction mixture. The enzyme kinetics were measured at 30°C for 10 minutes; fluorescence of the product was recorded at excitation and emission wavelengths of 380 and 460nm, respectively, using a Biotek Synergy MX plate reader. The kinetic data were derived by performing a non-linear least square fit of the experimental data using Michaelis-Menten equation and Graphpad Prism 7.

### Circular dichroism analysis

The far-UV circular dichroism (CD) spectrum of C105A-calpain was recorded using a Jasco J-715 CD spectropolarimeter equipped with a PTC 423S Peltier element. The protein concentration was 2μM in 20mM MOPS pH 7.5. The far-UV spectrum of the protein was recorded in a high-quality quartz cuvette of 1mm path-length at the wavelength from 260nm to 200nm, at scanning speed of 20nm/min, a response time of 2 seconds and a spectral bandwidth of 1nm. The final CD spectrum was the result of 3 accumulated scans.

The secondary structure content based on the CD spectrum was derived and compared to the deposited crystal structure of the calcium-free human m-calpain (PDB ID: 1KFU)[3] using the BeStSel web server (available at http://bestsel.elte.hu) [18]. The scaling factor was set to 0.1 in order to generate the best fitted data with the lowest possible normalized root-mean-square deviation (NRMSD) score.

### Interaction analysis of GST-hCSD1 and biotinylated-hCSD1 to calpain using bio-layer interferometry

To monitor interaction of calpain with calpastatin, we immobilized GST-hCSD1 and b-hCSD1 at a final concentration of 10 μg/ml to anti-GST and streptavidin (SA) biosensors, respectively. The *E*.*coli* lysate containing expressed C105AΔG2 was diluted 10x, 100x and 1000x and loaded to the hCSD1-immobilized sensors. As negative controls, we used the non-functionalized sensors that were dipped in the C105AΔG2 lysate for the association phase. In case of the anti-GST biosensor, a biosensor with immobilized GST was used as a second reference. The signal from these control samples was subtracted during the data processing. The experiments run at 30°C, at agitation of 1000 rpm, were recorded by the ForteBio data acquisition software version 7.1, and analyzed by the data analysis software of the same package.

### Bioinformatics sequence analysis

We assembled a comprehensive data set of 20 experimentally validated homologous amino acid sequences of CSDs by performing a PSI-BLAST against the non-redundant (NR) protein set of NCBI [19]. The query sequence was the first domain (residues 137–277; hCSD1) of the human calpastatin (UniProt ID: P20810). The data set of retrieved sequences covered the vertebrate taxonomic groups of mammals, birds and amphibians. We generated a multiple sequence alignment (MSA) with MAFFT and used the MSA as input for the sequence- and disorder conservation analysis carried out with the local version of the DisCons tool[20,21]. Briefly, DisCons calculates the position-specific sequence- and disorder conservation scores based on a multiple sequence alignment. DisCons was used with default parameters, namely predicting disorder with IUPred, and quantifying the sequence conservation with Jensen-Shannon divergence [21]; the maximum allowed fraction of gaps for a position was set to 0.6. The conservation trajectories were plotted using R.

## Results

### Selecting an eligible hCSD1 immobilization strategy

Initially, we chose glutathione S-transferase (GST) as an affinity-tag to immobilize GST-hCSD1 to a GSTrap column for various reasons, in particular because (i) the CAPN2-constructs contain a C-terminal his-tag; (ii) GST is a well-folded protein that is easily produced and does not interact with calpain; (iii) GST is unlikely to interfere with the interaction of hCSD1 with calpain; (iv) it is possible to remove the GST tag by thrombin cleavage to produce the calpain-calpastatin complex; and (v) GST can be used as a negative control for detailed interaction studies. The second choice was the chemical biotinylation of hCSD1 followed by its immobilization to streptavidin.

**Fig 1:**
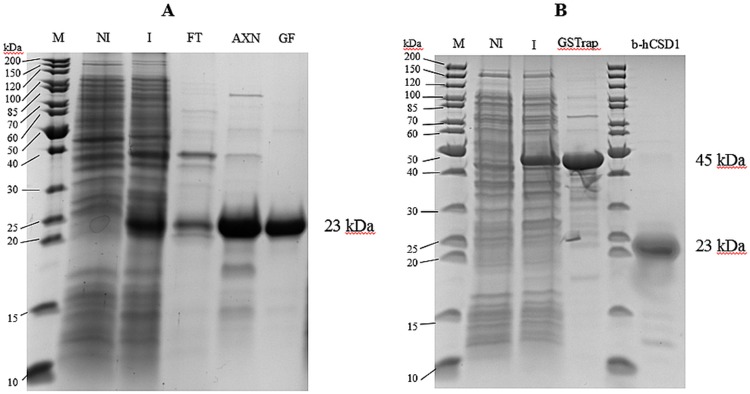
Expression and purification of (A) hCSD1, (B) GST-hCSD1 and b-hCSD1. NI: Non-induced; I: Induced; FT: Flow-through of hCSD1 lysate after anion exchange chromatography; AXN, GF: hCSD1 after anion exchange chromatography and gel filtration, respectively; GSTrap: GST-hCSD1 at 45kDa band after GSTrap affinity chromatography.

hCSD1 was easily produced with high purity (based on SDS-PAGE analysis) and in high quantity (Fig 1A). The protein appeared as a 23kDa band on an SDS-PAGE gel due to its aberrant electrophoretic mobility because of its disordered amino acid composition (see section ‘sequence analysis’), which has been reported in literature [1]. As expected, the GST-hCSD1 variant expressed well in *E*.*coli* and was purified by the GSTrap affinity chromatography (Fig 1B).

The chemical biotinylation of hCSD1 (b-hCSD1) was successful and yielded 2 mol biotin per mol of hCSD1. In addition, we verified the integrity of b-hCSD1 via SDSPAGE (Fig 1B).

**Fig 2:**
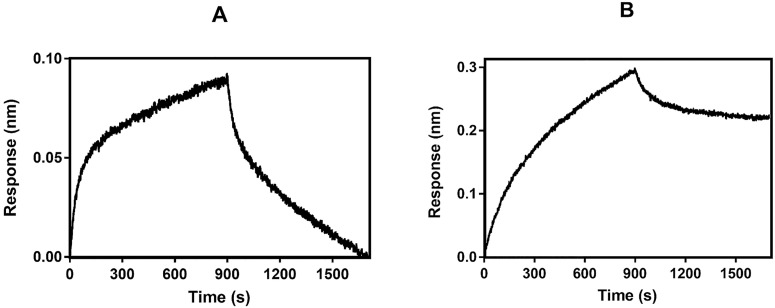
Binding kinetics of hCSD1 with C105AΔG2-containing lysate with 2 different immobilization strategies. Panel A and B show the binding of biotinylated-hCSD1 and GST-hCSD1, respectively, to the 1000-fold diluted lysate that contained recombinantly expressed C105AΔG2.

Even though the interaction of hCSD1 and calpain has been well described in literature using various techniques, we wanted to probe whether the GST-tag and biotinylation would hamper the binding capacity of hCSD1. Hence, we investigated whether immobilized hCSD1 is capable of capturing calpain from bacterial lysate using bio-layer interferometry (BLI). In both cases, the binding signals were recorded (Fig 2) and lead us to conclude that neither the biotinylation nor the GST-tag impedes the binding capability of hCSD1 to capture calpain from a biologically complex mixture. Yet, the dissociation phases of the binding curves (Fig 2) suggest that the binding of m-calpain to biotinylated hCSD1 is almost irreversible as compared to the GST-immobilized hCSD1 construct. This observation could either be explained by differences in protein flexibility due to the tag position (GST is N-terminally fused through a linker, while biotin is coupled to primary amines of lysine side chains), or by the physicochemical differences between the biosensors used. Nonetheless, since this calcium-mediated hCSD1-to-calpain binding can be abolished with EDTA to chelate calcium [22], we wish to exploit these binding characteristics to selectively isolate m-calpain.

As we found that the GST-tag and biotinylation do not hamper the binding of hCSD1 to calpain, our initial strategy comprised of the immobilization of hCSD1 with an N-terminal GST-tag followed by the application of the crude calpain-containing bacterial lysate in the presence of calcium to the immobilized GST-hCSD1 and finally the elution of the calpain heterodimer using a chelating agent like EDTA. Unfortunately, this initial strategy failed since GST and GST-hCSD1 were co-eluted with the target protein upon the application of EDTA. Although it might be possible that GST and/or GST-hCSD1 were proteolyzed by calpain activity, we established that the elution of GST by EDTA also occurs in the absence of calpain. For this reason, we decided to implement chemically biotinylated hCSD1 as our immobilization strategy.

### Production and purification of calpain

**Fig 3:**
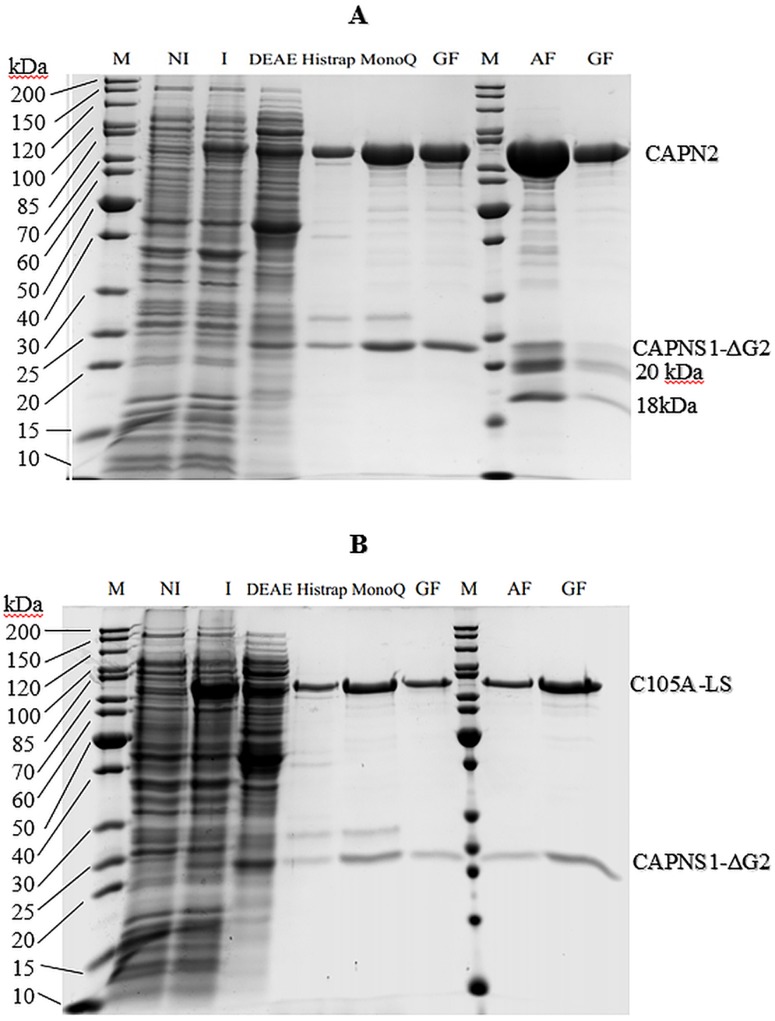
Expression and purification of WT- (A) and C105A- (B) calpain by conventional and AF methods. NI: Non-induced; I: Induced; DEAE, MonoQ: anion exchange chromatography using diethylaminoethyl and MonoQ column; Histrap: immobilized metal affinity chromatography; GF: gel filtration; AF: immobilized b-hCSD1 affinity chromatography.

Both WT and C105A-calpain expressed well in *E*.*coli* (Fig 3). It was observed that the solubility of the protein could be improved by expressing the protein at low temperature, preferably 16°C. Although it was previously reported that 5.8mg of purified m-calpain could be obtained from 1-liter *E*. *coli* culture [11], the maximum yield that we could reach was 1mg/L bacterial culture for WT-calpain and 0.4 mg/L bacterial culture for C105A-calpain by the conventional multistep purification method. By applying the affinity purification (AF) with b-hCSD1, we were able to increase the production yield at least 2-fold, in which 2mg of WT and 1mg of C105A-calpain were obtained and the production time was significantly reduced from 3 days to 1 day. Furthermore, despite applying only two steps instead of four, the sample purity was identical in case of the C105A-calpain as evidenced by SDS-PAGE (Fig 3B). In the presence of calcium, autolysis does not happen with inactive C105A-calpain, but the protein solution gradually shows the appearance of aggregates. This aggregation might prevent the interaction of C105A-calpain with the affinity column and might reduce the purification yield. Hence, it is necessary to speed up the purification process upon the addition of calcium. The situation is different with WT-calpain, which undergoes autolysis, two additional bands of the small subunit appeared at around 17kDa and 20kDa (Fig 3A). To confirm that these are autolysis products of the small subunit, one will need to run mass spectroscopy experiments, which has not been done within the framework of our study. This autolysis adds to the heterogeneity of the WT calpain sample (Fig 3A), which renders it less amenable for biophysical studies, yet it still useful for enzymatic or biological studies (e.g. characterization of calpain inhibitors) (see next section). The C105A inactive calpain mutant is purified as a highly homogeneous sample (Fig 3B) of sufficient quality for in depth biophysical studies.

### Characterization of the purified m-calpain for biophysical studies

**Fig 4:**
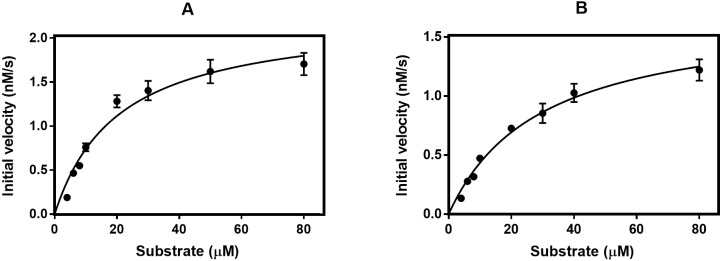
Michaelis-Menten plot of the WT calpain purified by conventional method (A) and affinity method (B).

We compared the performance of the two purification methods by analyzing the enzymatic activity of the calpain samples. The enzyme kinetic parameters of the purified WT-calpain were derived from the Michaelis-Menten plot (Fig 4) and summarized in Table 1. The data show that the affinity of the enzyme purified by AF to the SUC-LLVY-AMC substrate is lower than that of the conventionally purified one. Following that, the rate of the reaction and the specific activity of the affinity purified enzyme are decreased by about 20% as compared to the conventionally purified protease. This is probably due to the partial proteolytic degradation that we observed for the small subunit. Indeed, Elce and co-workers reported that the small subunit truncation could interfere with the formation of a stable and active heterodimer [23]. Likewise, Edmunds and coworkers (1991) showed that, when exposed to calcium, the 80kDa large subunit and the 30kDa small subunit are rapidly cleaved to 78 kDa and 18 kDa fragments and that this 78kDa/18kDa form of calpain retains 80% if its enzymatic activity [24]. Similarly, the observed small subunit degradation for the WT enzyme (Fig 3A) is likely to explain the reduction of the specific activity with 20% (Table 1) in our study.

**Table 1:** Comparison of the kinetic parameters of the enzyme purified by two methods.

	Km (μM)	Vmax (nM.s^-1^)	Kcat(s^-1^.10^−3^)	Kcat/Km (μM^-1^.s^-1^. 10^−3^)	Specific Activity (nmol.s^-1^.mg^-1^)
AF	29.32 ± 5.66	1.708 ± 0.233	12 ± 1	41.3 ± 2.6	0.112 ± 0.014
Conventional method	20.54 ± 2.28	2.262 ± 0.209	15 ± 2	73.2 ± 4.6	0.148 ± 0.015

**Fig 5:**
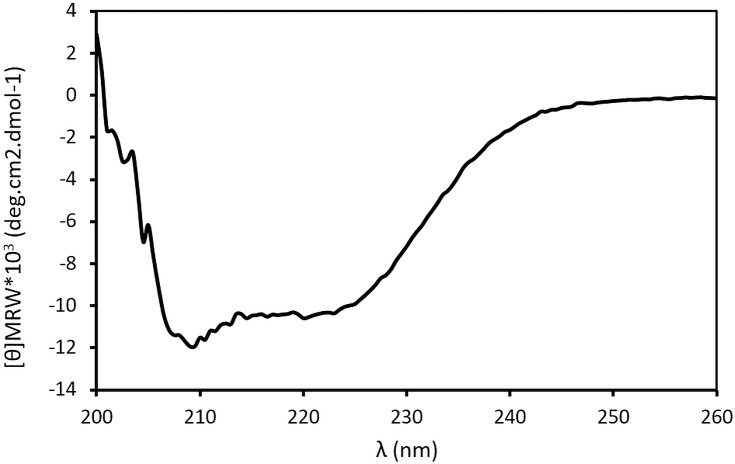
Far-UV CD spectrum of the C105A-calpain purified by AF method.

Nonetheless, for structural studies the inactive C105A calpain mutant has been preferred to avoid any heterogeneity arising from autoproteolysis [10]. As expected, the C105A-calpain showed no enzyme activity. Therefore, the quality of the purified C105A-calpain from AF was confirmed by far-UV CD spectroscopy (Fig 5). The secondary structure content estimated from the wavelength spectrum is in agreement with that derived from the crystal structure (Table 2). The experimental data was fitted (with an RMSD of 0.115 and an NRMSD of 0.02528) and showed that the protein structure contains a large portion of helix (about 30%) and β-strand (about 30%).

**Table 2:** Estimated secondary structure content (%) of the CD experimental curve (A) and the 1KFU.pdb crystal structure (B) using the BeStSel web server.

	A	B
Helix 1 (regular)	19.8	18.6
Helix 2 (distorted)	9.2	11.8
Antiparallel 1 (left-twisted)	2.7	0.4
Antiparallel 2 (relax)	9.3	5.1
Antiparallel 3 (right-twisted)	12.0	8.2
Parallel	5.9	0.4
Turn	10.4	11.3
Others	30.8	44.3

The fact that we observed a single elution peak at an apparent molecular weight of 125 kDa with an analytical gel filtration whereby SDSPAGE revealed the presence of both the large and small subunit, in combination with the CD-based secondary structure estimate of the C105A-calpain, confirmed that the protein was purified as a well-folded heterodimer that is suitable for biophysical studies (Fig 3B).

In addition, we could establish with BLI that the purified C105A-calpain from the AF method interacts with GST-immobilized hCSD1 with a *K*_D_ of 1.7 nM, which is in agreement with the K_D_-value reported in literature [5].

### Purification of the large and the small subunit of human m-calpain by the AF method

**Fig 6:**
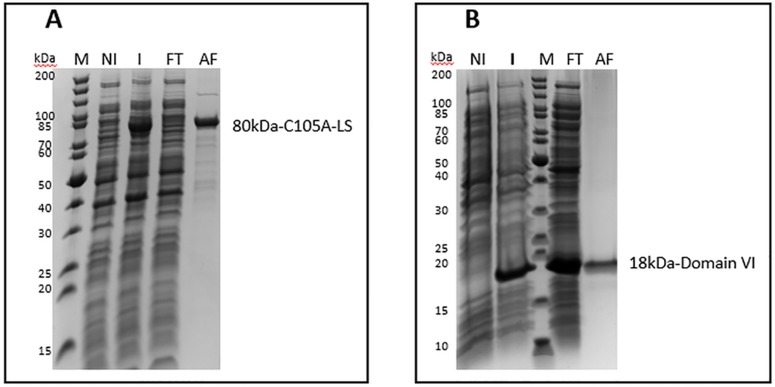
Coomassie-stained SDS-PAGE analysis of the calpain large subunit (A) and domain VI (B) that were purified by our new affinity method. M: Molecular weight marker proteins; AF: affinity chromatography; GF: gel filtration; NI: non-induced; I: induced; FT: flow through after affinity chromatography.

Herewith we also provide the evidence that the hCSD1-based affinity purification strategy can be used to capture fragments or individual domains of calpain. This is due to the modular structure of the binding motifs of hCSD1. To this end, we purified the 80 kDa large subunit (Fig 6A) and the 21 kDa domain VI (Fig 6B) of human m-calpain. In case of domain VI, we noticed that a considerable amount of protein remains in the flow-through fraction, which suggests that the column was saturated and was unable to trap more proteins. Nonetheless, our method also enables the single-step purification of individual subunits or domains of calpain.

### Sequence analysis of hCSD1 for general applicability of the new affinity purification strategy

**Fig 7:**
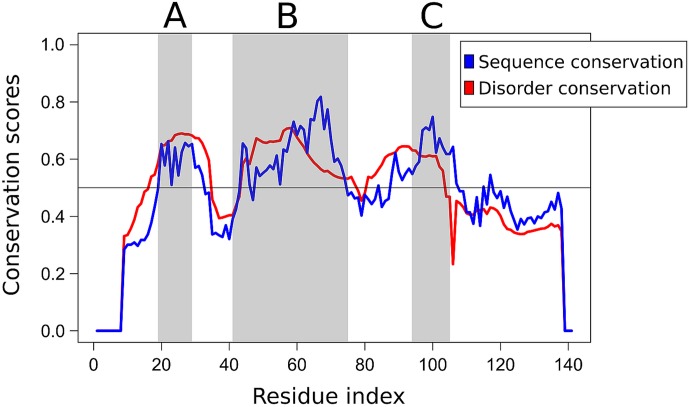
Sequence- and disorder conservation profile of CSDs. The conservation of both the amino acid sequence (blue) and structural disorder (red) of CSD was quantified by considering a comprehensive set of homologous vertebrate CSD sequences covering mammals, birds and amphibians. The two anchoring regions (A and C) and the inhibitory segment (B) are shaded in grey.

We also performed a comparative sequence analysis to provide indirect evidence for the specificity of the disordered binding regions of calpastatin and to assess the possible generality of the affinity principle developed. We collected a comprehensive dataset of homologous calpastatin domain sequences (CSD1 to 4), and with the help of DisCons [25] we quantified the conservation of both the amino acid sequence, and the structural disorder of the protein (Fig 7). Interestingly, the three binding segments of all CSDs (region A, B and C) are significantly more conserved across the investigated species than the linker regions between them, and disorder conservation is also peaking at these binding segments. Interestingly, the sequence is less conserved than disorder within the linker regions. This is in accordance with earlier studies that disordered binding regions, and specifically Molecular Recognition Features (MoRFs) have relatively higher sequence conservation than their disordered flanking regions. In contrast, sequence conservation is generally lower than the conservation of disorder at protein chains whose main functional role is to act as flexible linkers [25–27]. These findings demonstrate that the amino acid sequence of the binding segments of CSDs is highly conserved, which suggests that calpastatins of various species might be interchangeable in the purification of calpains.

## Discussion

Our work endorses the view that the conventional scheme of purifying recombinant human m-calpain is time consuming, labor intensive and requires different chromatographic resins. Despite a terminal His-tag, it requires at least 3 consecutive chromatographic steps to purify the protein, as a single IMAC step resulted in substandard quality protein samples. Other affinity purification schemes in literature either require pre-preparation steps such as antibody generation, or protein enrichment [12,28], or they have the risk of co-purifying other proteins in a calcium dependent manner [15]. Among them, Anagli and coworkers described a similar method to our newly devised approach to purify calpain from crude extracts of human erythrocyte and bovine kidney or heart by using calpastatin fragments [12]. So far, this method has not been used for purifying recombinant human m-calpain expressed in *E*. *coli* and it comprises an anion exchange chromatography before the peptide-affinity purification. Therefore, we have developed a new and simple affinity purification by taking advantage of the binding specificity of hCSD1.

Even though the BLI data confirmed that a GST-tag or biotinylation can be used for hCSD1 immobilization, we observed that the GST-hCSD1 co-eluted with the target protein in an EDTA dependent manner, implying that the interaction of GST-glutathione is disrupted by chelating agents (*i*.*e*., EDTA). Therefore, we resorted to chemical biotinylation of hCSD1 for immobilization, an approach that proved efficient for the single-step affinity purification. Although it was not directly observed by us, chemical biotinylation of hCSD1 might affect its binding properties because it occurs at the primary amines of the protein (i.e. the N-terminus and the lysine residues). A more controlled and elegant way to biotinylate hCSD1 would be to employ the BirA ligase in order to site-specifically modify the lysine of the avi-tag, a 15 amino acid peptide that can be fused to the target protein [29]. Another option would be to use the sortase labeling reaction to label the protein of interest with a biotinylated peptide [30]. Alternative immobilization strategies could rely on activated resins, such as such as the popular cyanogen bromide- or N-hydroxysuccinimidyl chloroformate-activated resins for the covalent attachment of the protein using the primary amine coupling.

One potential limitation to the approach that we present here may be manifested in the autoproteolysis of active calpain, as even an attempt to pre-incubate hCSD1 with calpain lysate did not remove autoproteolysis. Even so, the ability to purify human m-calpain variants (wild-type and inactive mutant) and its domains with a purity that is eligible for biological and biophysical studies by a single-affinity chromatography step is the major advancement thanks to our method. Furthermore, it enables to isolate calpain directly from biological samples, which offers new avenues for studying the enzyme under physiological conditions, for example in its native post-translationally modified form.

Since IDPs in general are susceptible to protease degradation [31], treatment with general protease inhibitors might be needed for the efficient use of IDPs in such a strategy. For the target protein (a proteolytic enzyme as in our case), this could be a complicating factor. Yet, we did not experience any problems by including general protease inhibitors during our purification. Additionally, the functionalized b-hCSD1 stationary phase could be reused for several subsequent chromatographies, at least when it was stored with 20% EtOH and protease inhibitors.

## Conclusion

In this study we have described a single-step affinity chromatography to purify recombinant human m-calpain, by exploiting the binding properties of its intrinsically disordered inhibitor, calpastatin. Compared to other established purification procedures, our method has several advantages: (i) it requires considerably less time investment (a single chromatographic step suffices); (ii) it can also be easily applied to purify individual calpain domains; (iii) the purity and the integrity of the purified protein is sufficient for structural biology studies; (iv) high throughput optimization of this method is possible through BLI (e.g. with different calpastatin variants, different screening conditions…); and (v) it enables rapid isolation directly from biological samples. The drawback of our method is the pre-activation of calpain and the subsequent autoproteolysis during purification in the case of the active enzyme. Although the specific activity of the WT enzyme purified by our newly devised method is somewhat lower than that resulting from the conventional purification method, it is definitely suitable for further structural and functional studies.
